# Mitochondrial MicroRNAs Contribute to Macrophage Immune Functions Including Differentiation, Polarization, and Activation

**DOI:** 10.3389/fphys.2021.738140

**Published:** 2021-11-03

**Authors:** Isabelle Duroux-Richard, Florence Apparailly, Maroun Khoury

**Affiliations:** ^1^IRMB, INSERM, Université de Montpellier, CHU Montpellier, Montpellier, France; ^2^Clinical Department for Osteoarticular Diseases, University Hospital of Montpellier, Montpellier, France; ^3^Laboratory of Nano-Regenerative Medicine, Faculty of Medicine, Universidad de Los Andes, Santiago, Chile; ^4^Cells for Cells and REGENERO, The Chilean Consortium for Regenerative Medicine, Santiago, Chile; ^5^IMPACT, Center of Interventional Medicine for Precision and Advanced Cellular Therapy, Santiago, Chile

**Keywords:** miRNA, mitochondria, macrophage, inflammmation, mitochondria transfer, microRNA, MitomiR

## Abstract

A subset of microRNA (miRNA) has been shown to play an important role in mitochondrial (mt) functions and are named MitomiR. They are present within or associated with mitochondria. Most of the mitochondrial miRNAs originate from the nucleus, while a very limited number is encoded by mtDNA. Moreover, the miRNA machinery including the Dicer and Argonaute has also been detected within mitochondria. Recent, literature has established a close relationship between miRNAs and inflammation. Indeed, specific miRNA signatures are associated with macrophage differentiation, polarization and functions. Nevertheless, the regulation of macrophage inflammatory pathways governed specifically by MitomiR and their implication in immune-mediated inflammatory disorders remain poorly studied. Here, we propose a hypothesis in which MitomiR play a key role in triggering macrophage differentiation and modulating their downstream activation and immune functions. We sustain this proposition by bioinformatic data obtained from either the human monocytic THP1 cell line or the purified mitochondrial fraction of PMA-induced human macrophages. Interestingly, 22% of the 754 assayed miRNAs were detected in the mitochondrial fraction and are either exclusively or highly enriched cellular miRNA. Furthermore, the *in silico* analysis performed in this study, identified a specific MitomiR signature associated with macrophage differentiation that was correlated with gene targets within the mitochondria genome or with mitochondrial pathways. Overall, our hypothesis and data suggest a previously unrecognized link between MitomiR and macrophage function and fate. We also suggest that the MitomiR-dependent control could be further enhanced through the transfer of mitochondria from donor to target cells, as a new strategy for MitomiR delivery.

## Mitochondria as Key Organelles in Macrophage Functions

Mitochondria are crucial cellular organelles that act as metabolic hubs and signaling platforms. Beyond the fateful endosymbiosis theory (Gray et al., 1999; Roger et al., 2017), mitochondria are multitasking, highly dynamic organelles that continually adjust their morphology, function, number and even their cellular host, in response to metabolic needs and environmental changes (Caicedo et al., 2017). Hence, their functions go beyond cellular ATP production and energy metabolism. In fact, they have indispensable roles in the immune system, especially in regulating macrophage polarization and responses to infection, tissue damage and inflammation.

## Mitochondria Triggering Macrophage Polarization

During pro-inflammatory activation of macrophages (so-called M1 polarization), aerobic glycolysis is activated (Van den Bossche et al., 2016; Liu Y. et al., 2021). This metabolic switch involves increased glucose uptake and pyruvate conversion to lactic acid, providing macrophages with the sufficient energy necessary for their anti-bactericidal activity (He et al., 2021). Moreover, the M1-type activation of macrophages inhibits mitochondrial OXPHOS and promotes mitochondrial nitric oxide (NO) production, thus preventing repolarization toward the anti-inflammatory M2 phenotype (Van den Bossche et al., 2016).

M2 macrophages have a complete tricarboxylic acid (TCA) cycle involving IL-4-mediated fatty acid oxidation (FAO) and mitochondrial energy metabolism as opposed to M1 macrophages (Jha et al., 2015). IL-4 induces ATP citrate lyase activation and oxidative phosphorylation (OXPHOS). In addition, it increases acetyl coenzyme A (acetyl-CoA) synthesis for histone acetylation, which is a key epigenetic regulator of the expression of several M2 marker genes. M2 macrophages maintain anti-inflammatory responses by obtaining most of their energy from FAO and oxidative metabolism (Qing et al., 2020).

IL-25 increases the mitochondrial respiratory capacity and oxygen consumption rate of macrophages and the production of NAD/NADH and ATP (Feng et al., 2017). This leads to the secretion of a large number of anti-inflammatory factors by macrophages for M2 polarization. Furthermore, the inhibition of NO production improves mitochondrial function and reprogramming into M2 macrophages (Mehla and Singh, 2019).

Mitophagy, a mechanism that selectively removes damaged mitochondria from cells, has also been related to macrophage polarization. During M1 polarization, mitophagy inhibition increases mitochondrial mass and decreases the expression of glycolysis-related genes and proinflammatory cytokines (Meng et al., 2021).

Although mitochondria are expected to act as a main target in macrophage regulation, the exact molecular mechanisms by which they regulate macrophage polarization is still not completely elucidated.

## Mitochondria Role in the Interplay Between Macrophage Metabolism and Host Defense Mechanism

Mitochondria are implicated in the tight interplay between host metabolic modifications and immune responses during bacterial infection (Ramond et al., 2019). Recently, it has been demonstrated that intracellular bacterial pathogens are able to modulate mitochondrial functions to maintain their replicative niche. Infection induces mitochondrial changes in infected macrophages, triggering modifications of the host metabolism that lead to important immunological reprogramming (Ramond et al., 2019). Indeed, stimulation by the bacterial toxin nigericin, or LPS, induced the NOD-like receptor family pyrin domain-containing 3 (NLRP3) to interacts with mitochondria via the mitochondria-associated adaptor protein MAVS, leading to ASC (Apoptosis-associated speck like protein) polymerization and downstream activation of caspase 1 and cytokine production (Subramanian et al., 2013). Assembly of the NLRP3 complex leads to the autocatalytic activation of caspase-1 and then the pro-inflammatory cytokines IL-1β and IL-18 (Kelley et al., 2019).

The uncoupling proteins (UCP), a subfamily of mitochondrial proteins, also plays a pivotal role in reprogramming macrophages during infection (Pecqueur et al., 2009). Upon LPS activation, macrophages downregulate UCP2 transcription, leading to a control of the mitochondria-derived reactive oxygen species (Emre and Nübel, 2010). Moreover, UCP2-(–/–) macrophages are more prompt to clear *S. typhimurium* intracellular infection (Arsenijevic et al., 2000).

## Involvement of MicroRNAs in the Regulation of Mitochondrial Processes

MicroRNA (miRNA) are evolutionarily conserved small non-coding RNAs, which regulate gene expression post-transcriptionally through mRNA degradation or translational inhibition (Bartel, 2004). Several studies demonstrate the presence of miRNAs in various organelles, including the nucleus, endoplasmic reticulum and the mitochondria. Mitochondria-associated endoplasmic reticulum membranes (MAM) are sites of contact where the endoplasmic reticulum domains interact with the mitochondria, facilitating communication between these two organelles (Gao P. et al., 2020). Recently, these sites have shown to contain a substantial number of miRNAs, which may be re-distributed in response to cellular stress and metabolic demands (Wang et al., 2020).

**FIGURE 1:**
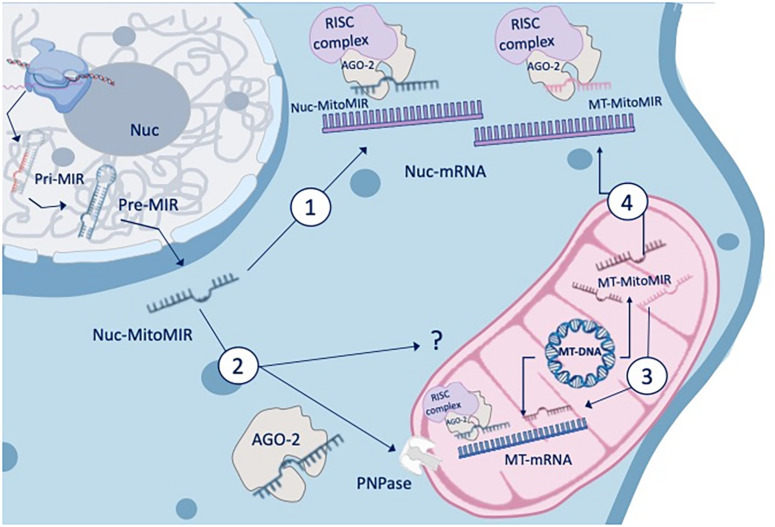
MitomiR and gene regulation. The three main types of miRNAs that are enriched in the mitochondrial fractions can either originate from the nuclear genome and mature in the cytoplasm to target cellular genes that modulate mitochondrial functions (nuc-MiR) or translocate into the mitochondria to target mitochondrial genes and functions (nuc-MitomiR), or be transcribed from the mitochondrial genome and target mitochondrial genes and functions (MT-MitomiR). The AGO2 protein, an endonuclease, has been reported to colocalize with mitochondria, and to be associated with small mtRNA. RNA and MitomiR import into mitochondria is facilitated by multi-subunit RNA import complex (RIC), through a pathway independent of protein import and recently, through AGO2 and the protein PNPase (poly nucleotide phosphorylase).

There are three main types of miRNAs enriched in the mitochondrial fractions (MitomiR): (1) They can originate from the nuclear genome and mature in the cytoplasm to target cellular genes that modulate mitochondrial functions (nuc-MiR), (2) they can translocate into the mitochondria to target mitochondrial genes and functions (nuc-MitomiR), or (3) they can be transcribed from the mitochondrial genome and target mitochondrial genes and functions (mt-MitomiR) (Bandiera et al., 2011; Figure 1).

The subclass of MitomiRs that originate from the nuclear genome that mature and regulate the expression of genes associated with mitochondrial pathways in the cytoplasm compartment is the most abundant. Numerous studies report and demonstrate their involvement in mitochondrial functions and activity, such as in apoptosis. Mitochondria play an important role in apoptosis progression, through the mitochondrial fission/fusion dynamics and the release of pro-apoptotic factors (Sheridan and Martin, 2010). For example, miR-200a-3p, miR-125b, miR-30 family, miR-485-5p, miR-195, and miR-140 regulate mitochondrial fission/fusion dynamics by targeting mitochondrial factors such as the mitochondrial fission factor (MFF) (Lee et al., 2017), the mitochondrial 18 kDa protein (MTP18) (Duroux-Richard et al., 2016), the dynamin-1-like protein (DRP1) (Li et al., 2010), the mitofusin (MFN) 1 and 2 (Li et al., 2014; Zhao et al., 2017; Purohit et al., 2019). As the heart is the organ with a perpetual high energy requirement, mitochondria occupy a large portion of cardiomyocytes, and are located between the myofibrils and just below the sarcolemma (Gustafsson and Gottlieb, 2008). Hence, strategic positioning and abundance of mitochondria ensure a highly efficient localized ATP delivery system to support contraction, metabolism, and ion homeostasis (Andrienko et al., 2003). The involvement of miRNAs in the energy control of mitochondria is therefore crucial in those cells. Indeed, in cardiomyocytes, upon apoptotic stimulation, miR-140 negatively regulates MFN1 and controls the release of endonuclease G, which participates in causing DNA fragmentation, and leads to apoptosis (Li et al., 2014). Following the anti-tumor drug Doxorubicin (DOX) treatment, miR-532-3p directly targets the caspase recruitment domain (ARC) and participates in DOX-induced mitochondrial fission and apoptosis. MiR-499-5p attenuates mitochondrial fission and DOX cardiotoxicity through p21 targeting (Wan et al., 2019).

## MitomiR and Cellular Functions

Apoptosis is also related to the pathogenesis of many diseases such as tumors, cardiovascular disorders and inflammatory responses, and miRNAs participate in the regulation of mitochondria-mediated apoptosis and metabolism. The miR-30 family regulates apoptosis through the targeting of the mitochondrial fission machinery leading to a suppression in the expression of p53 and its downstream target DRP1 (Li et al., 2010).

In human monocytes upon inflammatory signal, miR-125b attenuates mitochondrial respiration and promotes mitochondrial hyper-fusion through the silencing of both the BH3-only proapoptotic protein BIK and the pro-fission protein MTP18, leading to macrophage apoptosis (Duroux-Richard et al., 2016). In renal tubular cells, under hypoxia, MTP18 is silenced by miR-668, preserving mitochondria from fragmentation and cell death (Wei et al., 2018). In addition, it has been shown that miR-338 targets cytochrome c oxidase IV, a key nuclear-encoded protein within the electron transfer chain in mitochondria, which is involved in ATP production (Aschrafi et al., 2008).

The first evidence that miRNAs can be imported into mitochondria and regulate mtRNA expression was demonstrated in the mtDNA-lacking 206 ρ°cell line. Several nuc-MitomiR, such as miR-181c-5p and miR-146a-5p were identified in mitochondria and target mitochondrial RNAs and mitochondria-associated mRNAs encoded by nuclear genes (Dasgupta et al., 2015). In 2014, Das et al. (2014) demonstrated that miR-181c encoded in the nucleus but matured in the cytosol, translocates into the mitochondria regulating the expression of mitochondrial genes such as *mt-COX1* (known as mitochondrially encoded cytochrome c oxidase I). Using an *in vivo* systemic miR-181c delivery to the heart, enforced expression of miR-181c leads to an alteration in oxygen consumption, ROS production, matrix calcium and mitochondrial membrane potential of mitochondria isolated from cardiac cells (Das et al., 2014). More recently it has been shown that all members of the miR-181 family can alter the myocardial response to oxidative stress, by either targeting *mt-COX1* (miR-181c) or tensin homolog gene *PTEN* (miR-181a and miR-181b) (Das et al., 2017).

## Identification of Mitochondrial-Enriched MicroRNAs

To identify mitochondrial-enriched miRNAs, miRNome studies of mitochondrial and cytosolic RNA fractions from the same cells were performed. nuc-MitomiR signatures, such as miR-494, miR-1275 and miR-1974, were enriched in mitochondrial fractions (Bandiera et al., 2011). Subcellular trafficking and cellular dynamics of intracellular miRNA translocation depends on AGO2 levels and its status of phosphorylation. The AGO2 protein, an endonuclease shared across multiple species necessary for RNAi, has been reported to colocalize with mitochondria and to be associated with small mtRNA (Maniataki, 2005; Bandiera et al., 2011). In addition, it has been shown that RNA import into mitochondria is facilitated by multi-subunit RNA import complex (RIC), such as pathways independent of protein import (Mukherjee et al., 2007), co-localization of AGO2 and AGO3 (Bandiera et al., 2011), and recently, AGO2 and the protein PNPase (poly nucleotide phosphorylase) (Shepherd et al., 2017).

The functional RNAs encoded by the mitochondrial genome are mainly ribosomal RNAs (rRNAs) and transfer RNAs (tRNAs). The AGO2 protein has been shown to be associated with mitochondrial tRNAs in the cytoplasm. In addition, new types of RNA of mitochondrial origin have recently been identified. These are double-stranded RNAs encoded in mitochondria that are capable of triggering antiviral signaling in humans (Dhir et al., 2018). Interestingly, Bandiera et al. (2011) profiled the expression of miRNAs in mitochondrial fractions purified from HeLa cells and interrogated their genomics to better understand the molecular basis underlying their mitochondrial localization. They showed that miR-1974, miR-1977, and miR-1978 have a perfect match in the mitochondrial genome with two mitochondrial tRNA genes, *TRNE* and *TRNN*, and with a stretch of the mitochondrial rRNA sequence *RNR1* (Bandiera et al., 2011).

Thus, the post-transcriptional regulation of miRNAs directly into the mitochondria, or next to the mitochondria, especially in MAMs, allows the rapid expression of mitochondrial genome to be adjusted according to the metabolic conditions and demands of the cell. The importance of metabolism in deciding the fate of immune populations is very clear. Under conditions of stress, macrophages adjust their metabolism to meet the energy demand, necessary for their activation and functions.

## MitomiR Profiling of Mitochondria Extracted From Monocytic Cell Line

MiRNAs play pivotal roles in regulating macrophage functions, including differentiation, polarization, recruitment, and activation of inflammation (Self-Fordham et al., 2017). The ability of the mitochondria to modify cellular metabolic profile, thereby allowing an appropriate response, is crucial for the correct establishment of immune responses. However, little is known about mitomiR involvement when taking into account macrophage functions, such as differentiation. Thus, in this review, to provide novel insight into macrophage differentiation-specific mitomiR signatures and associated mitochondrial pathways, we performed a global miRNA profiling on extracted mitochondria and total cell content of human macrophages. The THP-1 monocytic cell line was treated with phorbol-12-myristate-13-acetate (PMA) to induce differentiation into macrophages.

**FIGURE 2:**
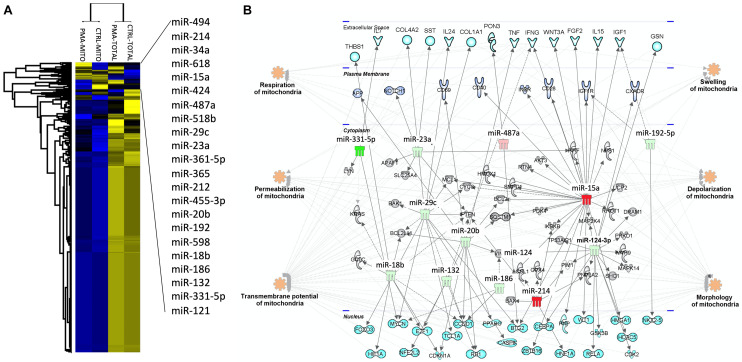
Macrophage mitomiRs signature: The human monocytic cell line THP-1 cells was differentiated into THP-1 derived macrophages with 40 ng/mL phorbol 12-myristate 13-acetate (PMA) over-night in RPMI 1640 Medium GlutaMAX^*TM*^ Supplement with 10% FBS at 37°C and 5% CO2. Total cellular and mitochondrial enrichment fractions were prepared at 4°C using a MACS Mitochondria kit (Miltenyi), and total RNA including miRNAs were extracted using miRNeasy kit (Qiagen). miRNA quantification was performed using the TaqMan^®^ Array Human MicroRNA Card Set v3.0 (Applied Biosystems), according to the manufacturer’s instructions. **(A)** Using Perseus software, a non-supervised hierarchical analysis of the distribution of total and mitochondria enrichment cell fractions based on relative log2 expression of 762 human miRNAs significantly modified by PMA compared to untreated controls. Yellow and blue represent the increase and decrease in expression, respectively. **(B)** miRNA-mRNA networks generated with Ingenuity Pathway Analysis (IPA) for the 22 macrophage-specific mitomiRs. IPA analysis was performed to investigate Mt-miRNA associated genes, filters were set to include experimentally observed or high-confidence-predicted miRNA-mRNA interaction partners, associated with mitochondria biological pathways. miRNAs were colored in red or green depending on whether they were upregulated or downregulated in mitochondria fractions following macrophage differentiation, respectively.

The mitochondrial fraction of the cells was enriched and purified using anti-TOM22 microbeads (Miltenyi Biotec). Briefly, 2 × 10^6^ were required to allow purification of the total fraction while 40 × 10^6^ cells were used for the isolation of the mitochondrial fraction. PMA-differentiated and undifferentiated THP-1 cells were centrifuged at 300 g at 4°C for 10 min, after homogenization in ice-cold lysis-buffer supplemented with antiprotease cocktail using 26G and 30G needles and 1 ml syringes (5 times round trips for each size). After checking that at least half of the effective lysis is achieved, the total cell fraction was obtained following centrifugation at 13,000 g for 5 min at 4°C. The obtained pellets were resuspended in 700 μl of Qiazol (Qiagen). In parallel, the mitochondrial cell fraction purification is carried out directly on the cell lysate by anti-TOM22 magnetic labeling (Miltenyi Biotec), according to the manufacturer’s instructions. After mitochondrial fraction elution, samples were centrifugated at 13,000 g for 5 min at 4°C, and mitochondria pellets resuspended in 700 μl of Qiazol for RNA extraction, using the miRNeasy kit (Qiagen). We performed the global miRNA profiling by considering 754 human miRNAs on extracted mitochondria and compared with total cell content. Total 500 ng RNA per sample were reverse transcribed to cDNA with Megaplex Human Pool A and B stem loop RT primers and TaqMan MicroRNA RT kit (Life Technology), and miRNA expression profiles were analyzed using the TaqMan^®^ Array Human MicroRNA Card Set v3.0, according to the manufacturer’s instructions.^1^ To address differences, the distribution of miRNA expression was visualized using unsupervised hierarchical clustering (Figure 2A). Data showed that 78% of miRNAs were very poorly detected in the mitochondrial fraction. The supervised hierarchical clustering analysis showed that 22 miRNAs are increased or decreased during macrophage differentiation in the mitochondrial compartment (Figure 2A, miRNAs name in right top panel). A miRNA profiling between total cell content and purified endoplasmic reticulum was also performed and showed no enrichment of the identified MitomiR (data not show), suggesting that these miRNAs are specific for mitochondria. Using ingenuity pathway analysis (IPA) tool,^2^ we examined which biological pathways were affected with the twenty-two identified MitomiRs and constructed mRNA-miRNA networks and miRNA-target gene interactions (Figure 2B). This analysis highlighted 9 (miR-331-5p, miR-18a-8p, miR-132, miR-29b-3p, miR-17-5p, miR-186-5p, miR-124-3p, miR-192-5p, and miR-23a-3p) and 4 (miR-494-3p, miR-214-3p, miR-16-5p, and miR-154-5p) MitomiRs were down- and up-expressed upon macrophage differentiation, respectively, and were potentially linked to key genes associated with regulation, permeabilization, morphology, depolarization, development, quantity, and transmembrane potential of mitochondria.

## MitomiR and Macrophage Functions

Interestingly, among these thirteen MitomiRs, miR-494 is probably one of the most described nuc-MitomiR. It is enriched in mitochondrial fractions and controls cell differentiation and mitochondrial functions in different cell types. It regulates mitochondrial biogenesis by silencing *mtTFA* and *Foxj3* during myocyte differentiation and skeletal muscle adaptation to physical exercise (Yamamoto et al., 2012). In ARPE-19 cells, a spontaneously arising retinal pigment epithelia (RPE) cell line, miR-494 is enriched in mitochondria and present in extracellular vesicles released by cells treated with rotenone to induce mitochondrial injury (Ahn et al., 2021). Thus, in addition to being the most frequently identified MitomiR (Geiger and Dalgaard, 2017), miR-494 is also involved in macrophage polarization and functions. Shao et al. (2020) showed that miR-494 enhances M1 macrophage polarization via *NRDP1* targeting in an intracerebral hemorrhage mice model. miR-494 also regulates inflammatory responses by targeting the phosphatase and *PTEN* and suppresses LPS-induced nuclear factor (NF)-κB signaling, in LPS-induced inflammation in mouse macrophages (Zhang et al., 2019). Interestingly, *PTEN* expression is strongly regulated by several miRNAs, including miR-214, which we found enriched in the mitochondrial fraction too. Down-regulation of *PTEN* expression leads to LPS-induced *AKT1* activation and inflammation (Fang et al., 2018). Furthermore, Lu et al. (2014) showed that the effect of macrophage polarization associated with the anti-cancer drug Norcantharidin on anti-hepatocellular carcinoma was due to increased miR-214 expression, which resulted in the inhibition of β-catenin signaling pathway. Recently, three studies demonstrated the key role of miR-214, miR-487a, and miR-124 in M2 macrophage polarization. The long non-coding RNA *NEAT1* promotes M2 macrophage polarization by sponging miR-214, which silences *B7-H3* (alias CD276), thus accelerating multiple myeloma progression via the JAK2/STAT3 signaling pathway (Gao Y. et al., 2020). MiR-487a is enriched in M2 macrophage-derived exosomes, which promote gastric cancer proliferation and tumorigenesis (Yang et al., 2021). Finally, miR-124 contributes to M2-type polarization of monocytic cells in normal conditions and during allergic inflammation (Veremeyko et al., 2013).

**FIGURE 3:**
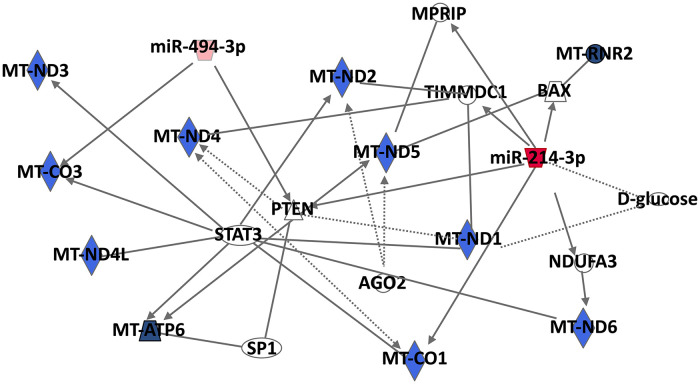
Mt-mRNA-miRNA networks generated with Ingenuity Pathway Analysis (IPA) for the two most described MitomiRs, miR-494, and miR-214. IPA analysis was performed to investigate genes associated with Mt-mRNAs. Filters were set to include either putative, experimentally observed or high-confidence-predicted miRNA-mRNA interaction partners. MiRNAs were colored in red depending on whether they were upregulated in mitochondria fractions following macrophage differentiation. The red color intensity corresponds to the fold change in miRNA expression in the mitochondria-enriched fraction of PMA-differentiated THP-1 macrophages compared to untreated THP-.

To study the mitochondrial signaling pathways and to shed light on the implication of MitomiRs on the regulation of mitochondrial functions, we performed an *in silico* analysis with the two most described MitomiRs, miR-494 and miR-214, whose expressions are up- and down-expressed, respectively, during monocyte differentiation into macrophage. Using IPA, we constructed a *mt*-mRNA-miRNA networks and a miRNA-target *mt*-gene interactions using putative miR-214 and miR-494 direct and indirect targets (Figure 3). Interestingly, miR-214-3p and miR-494-3p putatively target two *mt*-genes: the cytochrome C oxidase subunit I (*mt-CO1*, alias *COX1*) and subunit III (*mt-CO3*, *COX3*), respectively. Human cytochrome c oxidase (COX) is composed of 13 subunits, including three catalytic subunits I-III (*mt-CO1*, *mt-CO2*, and *mt-CO3*) encoded by mitochondrial DNA and ten nuclear-coded subunits by nuclear DNA (Kadenbach and Hüttemann, 2015). Furthermore, COX phosphorylation is described as having a strong influence on mitochondrial respiration and controlling macrophage function during inflammatory stimuli.

For example, the osteopontin, a cell attachment protein, that trigger a number of signal transduction pathways controlling survival, proliferation and migration of macrophages (Gao et al., 2007), decreases its expression in the presence of *mt-CO1* and *mt-CO3* in RAW264, murine macrophages, through a CD44-dependent transcriptional regulatory mechanism of the mitochondrial H strand (Gao et al., 2003). In macrophages, endotoxin-stimulated NO production inhibits cellular respiration and mitochondrial electron transport by inhibiting cytochrome c oxidase activity, and inhibits *mt-CO1* expression at the mRNA and protein levels, determining the host inflammatory response (Wei et al., 2002).

Finally, a RNA sequencing study of human macrophage subpopulation isolated from bronchoalveolar lavage fluid, aiming at deciphering macrophage plasticity in the lung microenvironment, revealed that macrophages with reduced expression of the classical surface markers M1 and M2 exhibit pro-inflammatory gene signatures, and overexpress 15 mitochondrial genes including *mt-CO3*, *mt-CO1*, *mt-RN*R2, *mt-ATP6*, *mt-ND1*, *mt-ND2*, and *mt-ND4L*, which may contribute to the pathogenesis and manifestations of inflammatory lung diseases such as chronic obstructive pulmonary disease (Takiguchi et al., 2021).

## MitomiR Transfer to Macrophage, a Novel Modulatory Strategy?

The clinical significance of this phenomenon was first assessed in a model of Lipopolysaccharide (LPS)-induced lung injury in which the intra-tracheal administration of mesenchymal stem/stromal cells (MSCs) to LPS treated mice was associated with the transfer of mitochondria to alveolar epithelium. MSCs triggered an increase in the concentration of ATP, metabolic activity, also an improvement in lung damage while reducing mortality in the diseased animals. Jackson et al. (2016) observed that mitochondria transferred from MSCs were able to increase the phagocytic capabilities of macrophages, increasing basal respiration and ATP turnover *in vitro* and *in vivo* in a murine model of acute respiratory distress syndrome (ARDS). The mechanism by which MSCs transferred mitochondria in this case was independent of *CNX43*, but related to the establishment of cellular highways (also known as tunneling nanotubules) between both cell types (Jackson et al., 2016). Interestingly, the artificial transfer of mitochondria from MSCs into macrophages, conveys the same metabolic and phagocytic improvement, evidencing a role for mitochondria in macrophage function (Liu D. et al., 2021). Recently, Yuan et al. (2021) showed in a diabetic nephropathy mice model, that mitochondrial transfer from MSCs to macrophages restricts inflammation and alleviates kidney injury via PGC-1α activation. These results represent strong evidence in favor of the hypothesis that the transfer of mitochondria from MSCs to immune active cells could play a role in the control of immune function mediated by MSCs (Court et al., 2020). However, mitochondrial transfer to macrophages is not specific for MSCs, Brestoff et al. (2021) demonstrated that adipocytes transfer their mitochondria to macrophages *in vivo*, mediated by heparan sulfates, which regulates white adipose tissue homeostasis, and plays a key role in obesity. In addition, recent studies showed that mitochondria from extracellular vesicles are involved in intercellular communication and immune regulation (She et al., 2021).

Also, mitochondria delivery can be applied following an artificial procedure, known mitoception (Caicedo et al., 2015), where extracted mitochondria from donor cells are transferred into recipient cells. Following this method, freshly isolated-mitochondria from MSCs could be transferred into immune cells including monocytes based on centrifugation and endocytosis. The “mitocepted” cells were able to increase their ATP production while shifting toward a glycolytic pathway. Thus, the process of mitoception allows a distinct analysis of the functional role of mitochondria, independently from the paracrine and cell-mediated effects from donor cells.

Mitochondrial transfer, whether mediated by cell-to-cell contact or through mitoception, presents a novel modulatory strategy through the delivery of mitomiR(s) to target cells, including macrophages (Jorgensen and Khoury, 2021).

## Hypothesis and Conclusion

Our *in silico* analysis identified a specific MitomiR signature associated with macrophage functions including differentiation, polarization, recruitment and activation of the inflammatory response. This MitomiR profiling correlated with gene targets within the mitochondrial genome or mitochondrial pathways. Our emitted hypothesis and data suggest a previously unrecognized link between MitomiR and macrophage functions and fate leading to a modulation of the downstream immune responses.

## Data Availability Statement

The datasets presented in this study can be found in online repositories. The names of the repository/repositories and accession number(s) can be found below: NCBI GEO GSE182255.

## Author Contributions

ID-R, FA, and MK drafted the manuscript. ID-R performed *in silico* analyses. All authors were involved in reading and editing the manuscript and approved the final version.

## Conflict of Interest

MK was the chief scientific officer of Cells for Cells and Regenero, the Chilean Consortium for Regenerative Medicine. The remaining authors declare that the research was conducted in the absence of any commercial or financial relationships that could be construed as a potential conflict of interest.

## Publisher’s Note

All claims expressed in this article are solely those of the authors and do not necessarily represent those of their affiliated organizations, or those of the publisher, the editors and the reviewers. Any product that may be evaluated in this article, or claim that may be made by its manufacturer, is not guaranteed or endorsed by the publisher.

## Footnotes

1
https://www.ncbi.nlm.nih.gov/geo/query/acc.cgi?acc=GSE182255

2
http://www.ingenuity.com
